# The Diversity of Karyotypes and Genomes within Section *Syllinum* of the Genus *Linum* (Linaceae) Revealed by Molecular Cytogenetic Markers and RAPD Analysis

**DOI:** 10.1371/journal.pone.0122015

**Published:** 2015-04-02

**Authors:** Nadezhda L. Bolsheva, Alexander V. Zelenin, Inna V. Nosova, Alexandra V. Amosova, Tatiana E. Samatadze, Olga Yu. Yurkevich, Nataliya V. Melnikova, Daria A. Zelenina, Alexander A. Volkov, Olga V. Muravenko

**Affiliations:** 1 Engelhardt Institute of Molecular Biology, Russian Academy of Sciences, Moscow, Russia; 2 Russian Federal Research Institute for Fisheries and Oceanography, Moscow, Russia; Huazhong university of Science and Technology, CHINA

## Abstract

The wide variation in chromosome number found in species of the genus *Linum* (2n = 16, 18, 20, 26, 28, 30, 32, 36, 42, 72, 84) indicates that chromosomal mutations have played an important role in the speciation of this taxon. To contribute to a better understanding of the genetic diversity and species relationships in this genus, comparative studies of karyotypes and genomes of species within section *Syllinum* Griseb. (2n = 26, 28) were carried out. Elongated with 9-aminoacridine chromosomes of 10 species of section *Syllinum* were investigated by C- and DAPI/С-banding, CMA and Ag-NOR-staining, FISH with probes of rDNA and of telomere repeats. RAPD analysis was also performed. All the chromosome pairs in karyotypes of the studied species were identified. Chromosome DAPI/C-banding patterns of 28-chromosomal species were highly similar. Two of the species differed from the others in chromosomal location of rDNA sites. B chromosomes were revealed in all the 28-chromosomal species. Chromosomes of *Linum nodiflorum* L. (2n = 26) and the 28-chromosomal species were similar in DAPI/C-banding pattern and localization of several rDNA sites, but they differed in chromosomal size and number. The karyotype of *L*. *nodiflorum* was characterized by an intercalary site of telomere repeat, one additional 26S rDNA site and also by the absence of B chromosomes. Structural similarities between different chromosome pairs in karyotypes of the studied species were found indicating their tetraploid origin. RAPD analysis did not distinguish the species except *L*. *nodiflorum*. The species of section *Syllinum* probably originated from a common tetraploid ancestor. The 28-chromosomal species were closely related, but *L*. *nodiflorum* diverged significantly from the rest of the species probably due to chromosomal rearrangements occurring during evolution.

## Introduction

The genus *Linum* L. (*Linaceae*) comprising more than 200 species, is characterized by a complex and questionable taxonomy that needs clarification. The wide variation in chromosome number which is found in species of the genus *Linum* (2n = 16, 18, 20, 26, 28, 30, 32, 36, 42, 72, 84) indicates that chromosomal mutations may have played an important role in the speciation of this taxon [1]. We have *previously* examined the members of sections *Linum* L. and *Adenolinum* (Reichenb.) Juz. (syn. *Linum*. *perenne* group) using a complex approach which had been developed for plants with small-sized chromosomes. The approach included chromosome elongation by use of DNA intercalators, chromosome banding, FISH and RAPD analysis [2]. It allowed us to compare the genome structures of the studied species at the chromosomal and molecular levels and make several predictions about relationships among the species as well as the genome origin of cultivated flax *Linum usitatissimum* L. [3–6]. The present article is a continuation of our molecular and karyological research of species of the genus *Linum*, which deals with the members of section *Syllinum* Griseb. This section comprises about 20 wild species with yellow or white flowers. Most members of the section are perennial shrubs, rarely herbs, with the exception of *Linum nodiflorum* L. which is an annual herb. The species of section *Syllinum* are common in Central and South-Eastern Europe, the Eastern Mediterranean, the Caucasus, Malaysia and Iran. Some of the species of the section are used as ornamental plants. Currently, the species of section *Syllinum* are considered to be potential bioproducers of biologically active substances, such as podophyllotoxin (one of the well-known bioactive naturally occurring *cytotoxic aryltetralin lignans*) served for synthesis of widely used anticancer drugs Etoposide, Etopophos and Teniposide [7, 8].

The taxonomy of *Linum* section *Syllinum* has been mostly based on morphological characters which have wide and continuous spectra of intraspecific variation; such variation often overlaps between species [9]. Accordingly, lots of species are morphologically similar. The use of molecular, genetic or biochemical markers did not permit differentiation among various species except *L*. *nodiflorum* [8, 10–14].Monochrome staining revealed different chromosomal numbers not only in karyotypes of various species of the section but also in karyotypes of conspecific samples except *L*. *nodiflorum*, which possessed a constant chromosome number 2n = 26 [15–18]. In addition, B chromosomes were revealed in the karyotype of *L*. *gyaricum* Vierh. belonging to section *Syllinum* [19]. Our early studies of several members from section S*yllinum* confirmed the presence of B chromosomes in their karyotypes. We found that regular chromosome complements (A chromosomes) always contained 28 chromosomes and the differences in the *number* of *chromosomes* revealed in the species were associated with the presence of B chromosomes [20–21]. At the same time, the identification of all the chromosomes in the complements and comparative genome analysis of the species were not carried out.

The present study aims to clarify the relationships among ten species of section *Syllinum* in order to better understand the phylogenetic relationships within the genus *Linum*. Accordingly, a comprehensive comparative karyotype analysis of the species from section *Syllinum* was conducted using a set of molecular cytogenetic markers. Genomic DNA of eight species of the section was also studied by RAPD analysis.

## Materials and Methods

### Plant material

The seeds of *Linum flavum* L. accession numbers LIN 99, LIN 1633, LIN 1527, LIN 97; *L*. *capitatum* Kit. ex Schultes accession numbers LIN 1903, LIN 1549; *L*. *campanulatum* L. accession number LIN 1760; *L*. *thracicum* Degen accession numbers LIN 1553, LIN 1764; *L*. *tauricum* Willd. accession numbers LIN 1611, LIN 1604; *L*. *elegans* Sprun. ex Boiss. accession number LIN 1652; *L*. *nodiflorum* L. accession numbers LIN 1877, LIN 1634 were obtained from the Gatersleben Genebank of the Institute of Plant Genetics and Crop Plant Research (Germany). The seeds of *L*. *capitatum* accession number K 6356 were obtained from All-Russian Flax Research Institute (Torzhok).

The seeds of *L*. *czernjajevii* Klokov (collected from wild populations of the Crimea) and *L*. *ucrainicum* (Griseb. ex Planch.) Czern. (collected from wild populations of the Donetsk region, Ukraine) were kindly supplied by Dr. O.M. Optasyuk, the M.G. Kholodny Institute of Botany, National Academy Sciences of Ukraine, Kyiv, Ukraine. The seeds of *L*. *dolomiticum* Borbás accession number 845 were kindly provided by Botanical Garden of Nantes, France.

Ten to twenty individual plants of each accession were taken for both chromosome and RAPD analysis. During seed germination, the seedings of *L*. *czernjajevii* and *L*. *dolomiticum* often died from undetermined infections. To avoid errors due to contamination of foreign DNA, we had to exclude these accessions from RAPD analysis.

### Preparation of metaphase chromosome spreads

The seeds were germinated in Petri dishes on moist filter paper at room temperature. To harvest elongated chromosomes, root tips (0.5 cm) were excised and treated overnight (16–20 h) in chilled water with 1 μg/ml 9-aminoacridine (Sigma, USA) according to the technique for elongating of small-sized chromosomes developed previously in our group [22]. After the pretreatment, the root tips were fixed in ethanol:acetic acid (3:1) for 3–24 h at room temperature.

For C-banding and Ag-NOR staining, chromosome spread preparations were obtained as described previously [6].

For FISH and DAPI/C banding, the root tips were transferred into 1% carmine solution in 45% acetic acid for 40 min. After that, the root tips were placed into a drop of 45% acetic acid on the microscopic slide, squashed under a cover slip and heated at 80°C for 1.5 min. The cover slips were removed after freezing in liquid nitrogen. Then, the slides were dehydrated in ethanol:acetic acid (3:1) and air-dried.

### C-banding

Chromosome slides were hydrolyzed in 0.2 M HCl for 3–5 min at 60°C, washed in running water for 10 min, dehydrated in ethanol and air-dried. The slides were incubated with a saturated solution of Ba(OH)_2_ for 6 min at room temperature, rinsed in 1 N HCl for 5–10 s and washed in running water for 15 min. Then, slides were incubated in 2×SSC at 60°C for 1 h, washed in running water for 15 min and air-dried. The slides were stained with 1.5% Giemsa solution (Merck, Germany) in 0.125 M Tris–HCl buffer (pH = 6.8) for 3–10 min.

### CMA staining

CMA staining was performed *according* to [23] with minor modifications. 50 μl of chromomycin A_3_ (Sigma, USA) solution (0.5 mg/ml chromomycin A_3,_ 5 mM MgC1_2_ in McIlvaine's buffer, pH 7.0) was dropped onto each slide and covered with a cover slip. The slides were incubated for 1–3 h at room temperature in a moist chamber in the dark. Then, cover slips were rinsed off with distilled water, and the slides were mounted in Vectashield medium (Vector *Laboratories*, UK).

### AgNOR staining

AgNOR staining was conducted *according to* [24]. After AgNOR staining slides were mounted in 0.125 μg/ml of DAPI (4',6-diamidino-2-phenylindole) (Serva, Germany) in Vectashield medium (Vector *Laboratories*, UK) for chromosome identification.

### FISH

FISH with rDNA probes was performed as described previously [6]. For FISH with a telomere repeat probe, the *Arabidopsis* type telomere repeat (TTTAGGG)_n_ was generated by PCR according to the protocol described by [25]. This probe was labelled with biotin-16-dUTP using the Biotin-Nick Translation Mix (Roche, Switzerland). For the detection of DNA sequences homologous to the telomere repeat, the chromosome slides were pretreated with 1 mg/ml RNase A (Roche, Switzerland) in 2×SSC (1×SSC is 0.15 M NaCl and 0.015 M sodium citrate) at 37°C for 1 h. The hybridization mixture containing 2×SSC, 40% formamide, 10% dextran sulphate and 2 ng/μl of biotinylated telomere DNA probe was used. The probe was hybridized overnight at 37°C. After hybridization the slides were washed twice with 0.1×SSC at 37°C for 10 min, followed by two washes in 2×SSC at 44°C for 5 min and a final wash for 5 min in 2×SSC at room temperature. The hybridization and wash conditions corresponded to 70% DNA homology. The biotin-labelled telomere repeat probe was detected with highly sensitive Tyramide Signal Amplification kit T-20932 (Life Technologies, USA).

### DAPI/C-banding

After *in situ* hybridization, slides were stained with 0.125 μg/ml of DAPI (Serva, Germany) in Vectashield mounting medium (Vector *Laboratories*, UK).

### Chromosomal analysis

The slides were examined using Olympus BX-61 epifluorescence microscope (Olympus, Tokyo, Japan). Images were captured with monochrome charge-coupled device camera (Cool Snap, Roper Scientific, Inc., Sarasota, FL, USA). Then they were processed with Adobe Photoshop 8.0 (Adobe Systems, Inc., San Jose, CA, USA). The chromosome images were measured with image analysis software “Videotest-Karyo 1.5” (IstaVideotest, St Petersburg, Russia). Based on the results of the measurements of 10 metaphase plates from 5 individual plants for each species, the chromosome ideograms were constructed.

### RAPD analysis

Genomic DNA was isolated using a Plant Genomic DNA Miniprep Kit (V-gene biotechnology, China). RAPD-PCR with standard decamer primers OPA10 and OPY02 was performed according to [26]. PCR-products were fractionated in 6% polyacrylamide gels. Then, the gels were stained with ethidium bromide solution and *scanned* using Typhoon 8600 variable-mode imager (Molecular dynamics, USA) at a resolution of 100 microns per pixel. The RAPD profiles were normalized against a molecular size scale. Cluster analysis as well as calculation of RAPD profile similarity according to Pearson correlation coefficient were carried out using Phoretix 1D Database software (Nonlinear Dynamics, UK).

## Results

### C-banding patterns of mitotic chromosomes

**Fig 1:**
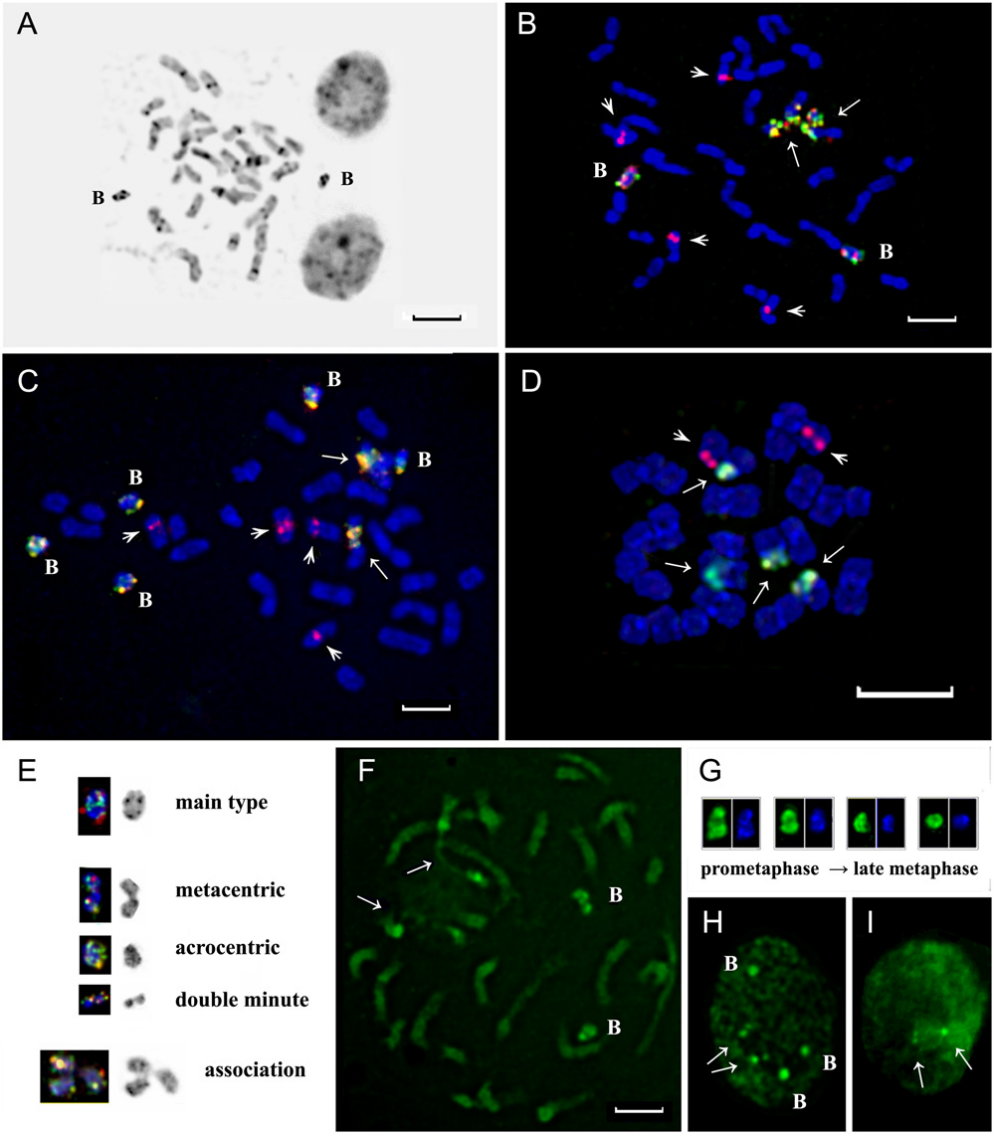
Chromosome spreads of different species of sect. *Syllinum*. C-banding pattern of *L*. *flavum* (A). Metaphase plates of *L*. *campanulatum* after FISH with rDNA probes having two (B) and five (C) B-chromosomes. A metaphase plate of *L*. *nodiflorum* after FISH with rDNA probes (D). Arrows point to satellite chromosomes with co-localized 26S (green) and 5S (red) rDNA loci; arrowheads point to chromosomes with 5S rDNA loci (red). Different morphological types of B-chromosomes (E). CMA staining of prometaphase chromosomes of *L*. *capitatum* (F). Arrows point to satellite chromosomes with two bright CMA bands revealed in the heterochromatic regions adjacent to NOR. DAPI (blue) and CMA (green) staining patterns of *B*-chromosomes at different stages of methaphase (G). CMA stained interphase nuclei of *L*. *capitatum* with (H) and without (I) B-chromosomes. Arrows point to small chromocenters formed by NORs. **B**—B-chromosomes. Scale bar—5 μm.

The karyotypes of *L*. *flavum*, *L*. *campanulatum*, *L*. *elegans*, *L*. *tauricum*, *L*. *thracicum*, *L*. *capitatum*, *L*. *dolomiticum*, *L*. *ucrainicum and L*. *czernjajevii* consisted of small—and middle-sized chromosomes (1.5–5.5 μm, after 9AMA treatment) which were metacentric or submetacentric. Analysis of C-banding patterns of this species showed that large C-bands were located in the pericentromeric chromosome regions and also in the regions adjacent to the nucleolus organizer region (NOR). Indistinct small telomeric and intercalary C-bands were occasionally revealed. B chromosomes were mainly stained with Giemsa more intensely than A chromosomes (Fig 1A). Intra- and interindividual variability of chromosome numbers in all the above-mentioned species (as a consequence of presence of B chromosomes in their karyotypes) has been revealed. C-banding patterns were not informative enough for reliable identification of all the individual chromosomes in the studied karyotypes.

### Chromosome identification and karyotype comparison

**Fig 2:**
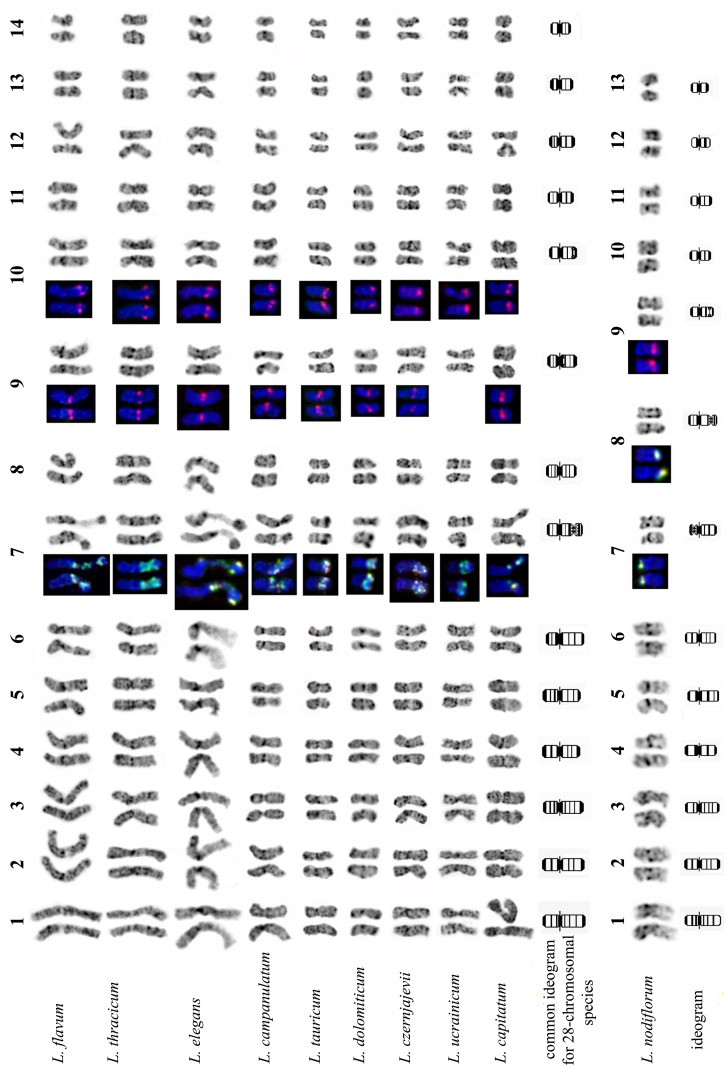
The karyotypes and chromosome idiograms of species from sect. *Syllinum*. Inverted DAPI/C-banding pattern (grey). Chromosomes are numbered according to their sizes. Localization of 26S (green) and 5S (red) rDNA revealed by FISH (coloured).

DAPI staining carried out after FISH, which included denaturation and renaturation of chromosomal DNA, revealed C-banding-like patterns (DAPI/C-banding patterns) in karyotypes of the members of section *Syllinum*. Optimization of a hydrolysis procedure (see ‘Material and Methods‘ section) significantly improved the quality of chromosome spreads as well as the resolution of DAPI/C-banding patterns in karyotypes compared with the usual C-banding technique. The combination of DAPI/C-banding and FISH with 26S and 5S rDNA probes allowed us to identify all the individual chromosomes in karyotypes of the species from section *Syllinum* (Fig 2, S1 and S2 Tables).

The chromosome analysis of nine species including *L*. *flavum*, *L*. *campanulatum*, *L*. *elegans*, *L*. *tauricum*, *L*. *thracicum*, *L*. *capitatum L*. *dolomiticum*, *L*. *ucrainicum* and *L*. *czernjajevii* showed that they have similar karyotypes. They are composed of fourteen chromosome pairs of a regular complement (A chromosomes) and a *variable number of* very small additional chromosomes (B chromosomes). The regular chromosome complements of all the nine species were identical in chromosome number, *centromeric index* and DAPI/C-banding pattern (Fig 2, S1 Table).

The substantial similarity in chromosome localization of rDNA loci was observed in all nine species. One co-localized 26S and 5S rDNA site was revealed in the secondary constriction region of the distal long arm of chromosomes 7. Furthermore, most of the studied species (*L*. *flavum*, *L*. *campanulatum*, *L*. *elegans*, *L*. *tauricum*, *L*. *thracicum*, *L*. *dolomiticum* and *L*. *capitatum*) possessed two separate 5S rDNA sites revealed in the proximal long arm of chromosome 9 and also in the distal long arm of chromosome 10 (Figs. 1B, 1C and 2). One very small 5S rDNA site was revealed occasionally on chromosome 9 in the karyotype of *L*. *czernjajevii*. In the karyotype of *L*. *ucrainicum*, one large 5S rDNA site was observed only on chromosome 10 (there was no 5S rDNA site on chromosome 9 at all) (Fig 2).

**Fig 3:**
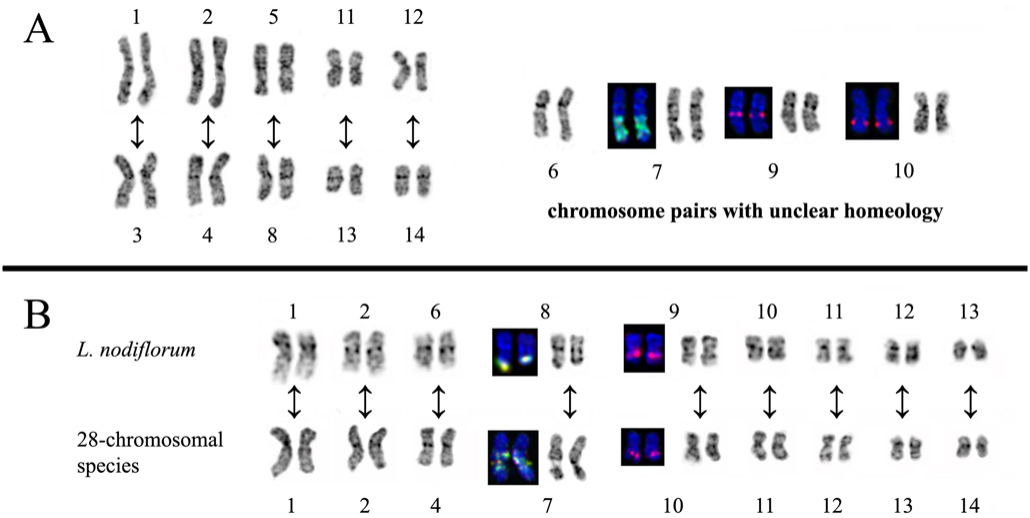
The structural similarities between different chromosome pairs. Putative homeologous chromosome pairs in karyotypes of 28-chromosomal species (A). Chromosome pairs of *L*. *nodiflorum* and 28-chromosomal species (B) similar in DAPI/C-banding pattern and localization of 26S (green) and 5S (red) rDNA.

Chromosome analysis also indicated that regular chromosome complements comprised two pairs of chromosomes which were similar in *centromeric index* and DAPI/C-banding pattern but were different in size. The similarity of DAPI/C-banding patterns was found between chromosome pairs 1 and 3, 2 and 4, 5 and 8, 11 and 13, 12 and 14 (Fig 3A). The data suggested that genomes of the studied species might have a tetraploid origin.

Aside from chromosomes of the regular complements (A chromosomes), small additional chromosomes (B chromosomes) were often observed in the karyotypes of the nine species. They were mainly stained with DAPI more intensely than A chromosomes. After FISH, B chromosomes were reliably identified as they contained multiple small co-localized 26S and 5S rDNA sites (Figs. 1B, 1C, 1E).

The variation in number of B chromosomes (ranged from 0 to 6) was revealed in root tip cells of the individual plants. In most cases, B chromosomes were small *submetacentric* chromosomes (1–1.5 μm). They were morphologically very similar to each other. After DAPI/C-banding, the largest bands were found in the pericentromeric region and in the distal long arm of B chromosomes. The described morphological structure was found in 90–95% of the B chromosomes revealed in karyotypes of *L*. *flavum*, *L*. *campanulatum*, *L*. *elegans*, *L*. *thracicum*, *L*. *capitatum*, *L*. *dolomiticum*, *L*. *ucrainicum* and *L*. *czernjajevii* (a main type of B chromosomes). But only 67% of B chromosomes of the main type were revealed in karyotypes of *L*. *tauricum*. Besides B chromosomes of the main type, the other types of morphology (metacentric, small acrocentric and dot chromosomes) were found in this species. All the types of B chromosomes were often involved in association (Fig 1E).

Unlike the nine above-described species of the section, the karyotype of *L*. *nodiflorum* carried 13 pairs of chromosomes which were smaller (2.0–4.0 μm) than chromosomes of the other species of the section, B chromosomes in *L*. *nodiflorum* were absent (Fig 2, S2 Table).

Chromosome DAPI/C-banding pattern allowed us to identify all 26 chromosomes in the karyotype of *L*. *nodiflorum* (Fig 2). Co-localized 26S and 5S rDNA sites were detected on small satellites and secondary constrictions of two middle-sized chromosomes (chromosomes 7 and 8). 5S rDNA site was revealed on chromosome 9 (Figs. 1D and 2).

Though rather small sizes, nine pairs of chromosomes of *L*. *nodiflorum* were morphologically similar to chromosomes of the other species of the section (Fig 3B).

### Distribution of GC-rich chromatin along chromosomes and in cell nuclei revealed by CMA staining

After CMA staining, bright bands in the area adjacent to the nucleolus organizer region (NOR) of the satellite chromosomes were revealed in karyotypes of all the species. In addition, B chromosomes in karyotypes of 28-chromosomal species bearing one bright band in the middle of the short arm and two bands in proximal and distal regions of the long arm were detected (Figs. 1F, 1G). Such bright CMA staining was mostly due to the presence of GC-rich ribosomal genes in NORs and on B chromosomes. Compared with A chromosomes, B chromosomes were tightly compacted during *all the stages of the cell cycle*. In metaphase, the length of B chromosomes decreased slightly as compared with prophase (Fig 1G). After CMA staining of interphase nuclei of the plants possessing B chromosomes, two-four small chromocenters adjacent to a nucleolus as well as large chromocenters mostly located *close to* the *nuclear periphery* (far from the nucleolus) were observed (Fig 1H). In contrast, large chromocenters were not found in the interphase nuclei of the plants which did not possess B chromosomes (Fig 1I).

### Detection of active NORs by Ag-NOR-staining

**Fig 4:**
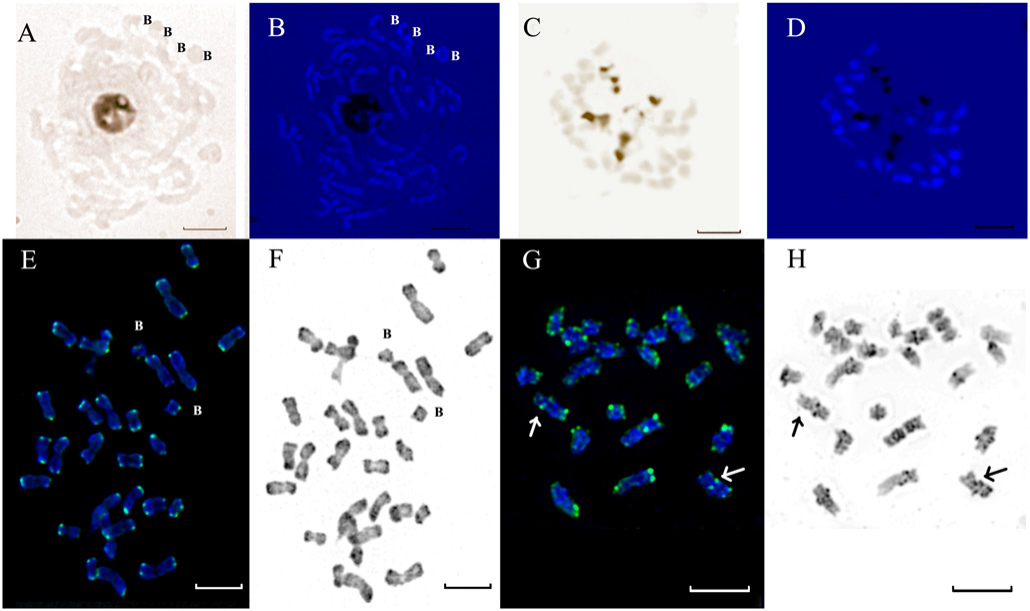
AgNOR staining and localization of telomeric repeats. AgNOR staining.of metaphase chromosomes of *L*. *flavum* (A) and *L*. *nodiflorum* (C). Superposition of AgNOR (dark) and DAPI staining (blue) of the same metaphase plates (B, D). Localization of telomeric repeats (green) on metaphase plates of *L*. *flavum* (E) and *L*. *nodiflorum* (G) revealed by FISH. Inverted DAPI staining (grey) of the same metaphase plates (F, H). Arrows point to intercalary loci of telomere repeats on chromosomes 3 of *L*. *nodiflorum*. **B**—B-chromosomes. Scale bar—5 μm.

For investigating the level of transcriptional activity of the revealed rDNA sites, the examined species were studied using Ag-NOR staining. It was found that the secondary constriction of chromosome 7 in karyotypes of *L*. *flavum*, *L*. *campanulatum*, *L*. *elegans*, *L*. *tauricum*, *L*. *thracicum*, *L*. *capitatum*, *L*. *dolomiticum*, *L*. *ucrainicum* and *L*. *czernjajevii* was stained by silver nitrate. At the same time, B chromosomes were not stained by silver nitrate (Figs. 4A, 4B) suggesting that they comprised no transcriptionally active rRNA genes. In metaphase of *L*. *nodiflorum*, two pairs of chromosomes were silver stained (Figs. 4C, 4D).

### Chromosome localization of telomeric repeats

FISH-analysis showed that the telomere repeat sequence was localized on chromosome ends in karyotypes of 28–chromosome species of the section (Figs. 4E, 4F). In addition to the terminal sites, in the karyotype of *L*. *nodiflorum* one intercalary site of the telomere repeat was revealed in the median region of the long arm of chromosome 3 (Figs. 4G, 4H).

### Genomic diversity studied by RAPD—analysis

**Fig 5:**
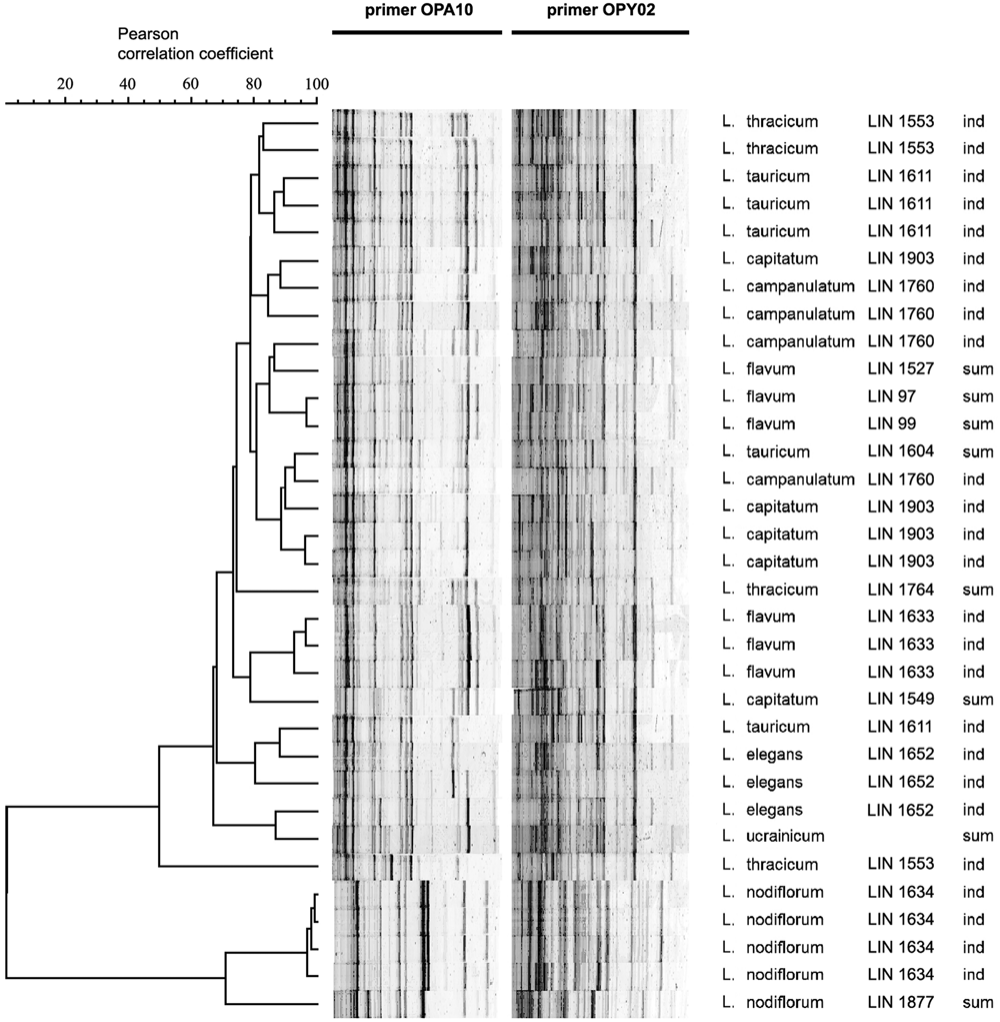
The UPGMA dendrogram of genomic relationships between species within sect. *Syllinum* and RAPD spectra obtained with POA10 and POY02 primers. “ind”—DNA obtained from individual plant; “sum”—bulk DNA obtained from ten individual plants.

For estimating the value of intergenomic divergence of species from section *Syllinum*, a variant of RAPD method based on the use of polyacrylamide gel electrophoresis for the separation of PCR products was performed. The employed technique, which revealed a lot more electrophoretic bands in comparison with the use of agarose gel electrophoresis, allowed us to use only two PCR primers for genomic analysis. The total number of the bands amounted to 117, and 101 of which were polymorphic. The number of fragments in the individual RAPD profiles ranged from 41 to 57. The UPGMA dendrogram of individual RAPD profile similarities of the species of section Syllinum is illustrated in Fig 5. As the dendrogram shows, the species from section *Syllinum* formed two main clusters. The first cluster comprised only the accessions of *L*. *nodiflorum*. The second large cluster combined the 28-chromosomal species *L*. *flavum*, *L*. *capitatum*, *L*. *campanulatum*, *L*. *thracicum*, *L*. *tauricum*, *L*. *ucrainicum* and *L*. *elegans*. Their RAPD spectra were rather similar (Pearson correlation coefficient r ≥ 55%). Inside this large cluster, the DNA samples of the plants belonging to one accession often formed subclusters. Occasionally, the RAPD spectra of individual plants were comparatively more like the RAPD spectra of other species than to the spectra of the rest plants of its own accession (such as in *L*. *thracicum* accession LIN 1553). The DNA samples of the plants belonging to different accessions of one species might cluster together, for instance, it was found in three accessions of *L*. *flavum* LIN 97, LIN 99 and LIN 1527. However, they might be at a considerable distance from each other (e.g., the fourth accession of *L*. *flavum* LIN 1633 or two accessions of *L*. *tauricum* LIN 1604 and LIN 1611).

## Discussion

### Comparative karyological analysis of species of sect. *Syllinum* and other sections of the genus *Linum*

The analysis of C- and DAPI/C-banding patterns in karyotypes of the species belonging to section *Syllinum* showed that the largest heterochromatic bands were located in the pericentromeric chromosome regions and also in the regions adjacent to the nucleolus organizers. The telomeric and intercalary C-bands were much smaller. A similar distribution of heterochromatin along chromosomes was previously revealed in karyotypes of species from sections *Linum* and *Adenolinum* [3, 6] as well as in a number of plant species having small-sized chromosomes [27]. At the same time, the chromosomes of species of section *Syllinum* differed markedly (in size, DAPI/C-banding pattern as well as in chromosome localization of ribosomal genes) from the chromosomes of earlier examined members of sections *Linum* and *Adenolinum* [3, 6]. The absence of karyological similarities between the yellow-flowered species of section *Syllinum* and the blue-flowered species of sections *Linum* and *Adenolinum* is probably due to their phylogenetic remoteness which has been found by recent molecular phylogenetic studies [13, 28]. The yellow-flowered species of sections *Linastrum* and *Cathartolinum* are still poorly studied by molecular cytogenetic techniques with the exception of two closely related species *L*. *suffruticosum* L. and *L*. *tenuifolium* L. of sect. *Linastrum* [4]. Both species also differ significantly from other species of sect. *Syllinum* in their karyotype structure. They have nine pairs of chromosomes, five of which bear 45S rDNA loci and one pair bears 5S rDNA locus. The only common feature of karyotypes of *L*. *suffruticosum* and *L*. *tenuifolium* and species of sect. *Syllinum* is predominant localization of heterochromatin in the pericentromeric areas.

### Karyological relationships within sect. *Syllinum*

Comparison of the karyotypes of *L*. *flavum*, *L*. *campanulatum*, *L*. *elegans*, *L*. *tauricum*, *L*. *thracicum*, *L*. *capitatum*, *L*. *dolomiticum*, *L*. *ucrainicum and L*. *czernjajevii* revealed high similarity in DAPI/C-banding pattern of their chromosomes. Chromosomal localization of ribosomal genes in seven species was identical. But the 5S rDNA signal on chromosome 9 in karyotypes of two species *L*. *czernjajevii* and *L*. *ucranicum* was very weak (or was not revealed at all), that was probably due to *copy-number reduction* of 5S ribosomal genes in the locus. It should be emphasized that both of the species *L*. *czernjajevii* and *L*. *ucranicum* are endemic forms of South-Eastern Europe where their areas overlap. The two species are well-adapted to chalky soil; they are morphologically quite similar and differ mostly in leaf indumentum. Plants having intermediate morphology were also found in the overlapping areas of the species [29–32]. All the data indicate that the two species are closely related.

We found that karyotypes of 28-chromosomal species of section *Syllinum* comprised five pairs of chromosomes which were different in length but similar in banding pattern. Probably, these species were meso-tetraploids, and the chromosomes having the same banding pattern were homeologous. The putative homeologous chromosomes in karyotypes of the members of section *Syllinum* were not completely identical. They differed in length and occasionally in size of some DAPI/C-bands. Therefore, the species of the section were most likely allotetraploids. But, the *differences in morphology of the* homeologous chromosomes could also appear during the process of diploidization of an autotetraploid. *In our previous work*, we had already detected morphologically similar karyotypes of tetraploid forms from section *Adenolinum* which differed in chromosome size [6]. The cause of this phenomenon is not clear, and it might reflect the common r*egularities* of polyploid formation.

B chromosomes had been previously detected in karyotypes of the members of section *Syllinum* [19–21]. In the present paper, we revealed that the structure and morphology of these supernumerary chromosomes were similar to the B chromosomes of other organisms. They were the smallest chromosomes in the karyotype which almost completely consisted of heterochromatin that remained condensed during all stages of the cell cycle and contained multiple loci of ribosomal genes. It should be noted that ribosomal genes were frequently found in B chromosomes [33] and such genes could be active or inactive [33–36]. We found that B chromosomes in karyotypes of the species of section *Syllinum* were not stained by silver nitrate and, accordingly, their 45S rRNA genes were not transcriptionally active. Structural polymorphism of the B chromosomes as well as intra-individual mosaicism in their number revealed in the studied species was not uncommon among other plant species having B chromosomes. It was suggested that the polymorphic variants of B chromosomes were *caused by* chromosome aberration, and the variation in number of B chromosomes, described in the cells of the same individual, was the result of the irregular disjunction of B chromosomes in mitosis [33, 37, 38].

The high similarity of karyotypes of 28-chromosomal species from section *Syllinum* confirmed their close relationships. We had previously described the karyological and the genomic similarities within some taxonomic groups of the genus *Linum* such as species of section *Adenolinum*, 16-chromosomal and 30- chromosomal species of section *Linum* [3, 6, 39].

The karyotype of *L*. *nodiflorum* was distinguished from the rest of the species of the section by its chromosome size and number (2n = 26) and also by absence *of* B chromosomes, and our results coincided with data obtained earlier [15, 18]. We also found that in contrast to the 28-chromosomal species there were two satellite chromosomes in the karyotype of *L nodiflorum*. However, the detailed comparative analysis revealed the similarities in structure of nine pairs of chromosomes in karyotypes of *L*. *nodiflorum* and 28-chromosomal species that confirmed their close relationships. At the same time, a karyotype of *L*. *nodiflorum* underwent a significant reorganization. In particularly, the intercalary site of the telomere repeat detected in chromosome 3 of *L*. *nodiflorum* could be a result of chromosome fusion, which then led to a reduction in number of chromosomes (from 28 to 26) in its karyotype.

### Genomic diversity of the species of sect. *Syllinum* revealed by DNA fingersprinting

The relationships of the species from section *Syllinum* revealed by chromosome analysis were confirmed by the results of RAPD analysis. Within the group of 28-chromosomal species, the specimens of one species were not always differentiated into isolated clusters but often were interspersed with the specimens of other species. Thus, the level of intraspecific genomic diversity of 28-chromosomal species was comparable to the level of interspecific differences.

For this reason, the distinguishing of different species within the group of 28-chromosomal species was impossible. In contrary, *L*. *nodiflorum* was genetically remote from the rest of the species of section *Syllinum*, and its accessions formed a clearly isolated cluster. The same results were obtained earlier by AFLP analysis which revealed significant differences between *L*. *nodiflorum* and other species of the section, but detected no differences among the five 28-chromosomal species [12].

## Conclusions

Our research shows that the species of section *Syllinum* probably originated from a common tetraploid ancestor. During evolution, the divergence of the ancestor of *L*. *nodiflorum* from the major phylogenetic branch of the section (28-chromosomal species) occurred. This divergence could be caused by chromosomal rearrangements which led to reproductive isolation of the ancestor of *L*. *nodiflorum* from the other members of the section. In contrast, the studied 28-chromosomal species of the section diverged slightly, perhaps more recently, and their reliable differentiation by molecular and karyological markers used in the present study was not possible.

## Supporting Information

S1 Table Structural and morphological characters of chromosomes of 28-chromosomal species of sect. *Syllinum*. “centromeric index”—the ratio of the length of the short arm of the chromosome to that of the total chromosome; “s.d.”—standart deviation; “S”—small; “M”—middle; “L”—large.
^a^—in most species exept *L*. *ucranicum* and some plants of *L*. *czernjajevii*.
(DOCX)

S2 Table Structural and morphological characters of chromosomes of *L*. *nodiflorum*. “centromeric index”—the ratio of the length of the short arm of the chromosome to that of the total chromosome; “s.d.”—standart deviation; “S”—small; “M”—middle; “L”—large.
(DOCX)
