# Supplementary material for: The Diversity of Karyotypes and Genomes within Section Syllinum of the Genus Linum (Linaceae) Revealed by Molecular Cytogenetic Markers and RAPD Analysis
Source: PLoS One. 2015 Apr 2;10(4):e0122015. doi: 10.1371/journal.pone.0122015 (PMC4383504; doi:10.1371/journal.pone.0122015)
Supplement: S1 Table — “centromeric index”—the ratio of the length of the short arm of the chromosome to that of the total chromosome; “s.d.”—standart deviation; “S”—small; “M”—middle; “L”—large. a—in most species exept L. ucranicum and some plants of L. czernjajevii. (DOCX) [file pone.0122015.s001.docx]

**S1 Table. Structural and morphological characters of chromosomes of 28-chromosomal species of sect. *Syllinum*.**

| **chromosome** | **mean**  **chromosome**  **length**  **+ s.d., (µm)** | **centromeric**  **index**  **+ s.d., (%)** | **DAPI/C - bands** | | | | | **rDNA**  **sites** |
| --- | --- | --- | --- | --- | --- | --- | --- | --- |
|  |  |  | **short arm** | | **centromeric**  **band**  **(size)** | **long arm** | |  |
|  |  |  | **telomeric**  **band**  **(size)** | **intercalaric**  **bands**  **(number)** |  | **intercalaric**  **bands**  **(number)** | **telomeric**  **band**  **(size)** |  |
| **1** | **5.5**  + 1.5 | **40.0**  + 3.7 | M | 2 | M | 3 | M |  |
| **2** | **5.4**  + 1.4 | **43.7**  + 3.5 | M - L | 1 | L | 2 | M |  |
| **3** | **5.2**  + 1.3 | **39.6**  + 3.0 | M | 2 | M | 3 | M |  |
| **4** | **5.0**  + 1.2 | **44.8**  + 3.8 | M - L | 1 | L | 2 | M |  |
| **5** | **4.9**  + 1.2 | **43.2**  + 3.2 | M | 2 | M | 2 | M |  |
| **6** | **4.9**  + 1.1 | **36.6**  + 3.6 | M | 1 | L | 2 | S - M |  |
| **7** | **4.8**  + 1.5 | **37.3**  + 4.7 | S - M | 1 | M - L | 3 | M | 5S + 45S rDNA colocalized sites  in the distal region of the long arm |
| **8** | **3.9**  + 0.8 | **44.3**  + 3.3 | M | 2 | M | 2 | M |  |
| **9** | **3.9**  + 0.7 | **37.2**  + 3.0 | M - L | 1 | L | 2 | M | 5S rDNA site in the  proximal region of the long arm^a^ |
| **10** | **3.6**  + 0.6 | **37.9**  + 3.6 | M | - | M - L | 2 | S | 5S rDNA site in the  distal region of the long arm |
| **11** | **3.4**  + 0.4 | **46.8**  + 3.5 | M | 1 | M | 1 | M |  |
| **12** | **3.4**  + 0.5 | **39.8**  3.1 | M - L | 1 | L | 1 | M |  |
| **13** | **3.0**  + 0.4 | **39.4**  + 3.4 | M | - | L | 1 | L |  |
| **14** | **2.7**  + 0.3 | **45.3**  + 3.8 | S - M | - | M | 1 | M |  |
| **B** | **1.5**  + 0.4 | **32.3**  + 3.3 | S | - | L | 1 | M - L | multiple 5S + 45S rDNA colocalized  sites distributed along the chromosome |

“centromeric index” – the ratio of the length of the short arm of the chromosome to that of the total chromosome; “s.d.” – standart deviation; “S” – small; “M” – middle; “L” – large.

^a^ - in most species exept *L. ucranicum* and some plants of *L. czernjajevii*.
