# Supplementary material for: The Diversity of Karyotypes and Genomes within Section Syllinum of the Genus Linum (Linaceae) Revealed by Molecular Cytogenetic Markers and RAPD Analysis
Source: PLoS One. 2015 Apr 2;10(4):e0122015. doi: 10.1371/journal.pone.0122015 (PMC4383504; doi:10.1371/journal.pone.0122015)
Supplement: S2 Table — “centromeric index”—the ratio of the length of the short arm of the chromosome to that of the total chromosome; “s.d.”—standart deviation; “S”—small; “M”—middle; “L”—large. (DOCX) [file pone.0122015.s002.docx]

**S2 Table. Structural and morphological characters of chromosomes of *L. nodiflorum*.**

| **chromosome** | **chromosome**  **length**  **+ s.d., (µm)** | **centromeric**  **index**  **+ s.d., (%)** | **DAPI/C - bands** | | | | | **rDNA**  **sites** |
| --- | --- | --- | --- | --- | --- | --- | --- | --- |
|  |  |  | **short arm** | | **centromeric**  **band**  **(size)** | **long arm** | |  |
|  |  |  | **telomeric**  **band**  **(size)** | **intercalaric**  **bands**  **(number)** |  | **intercalaric**  **bands**  **(number)** | **telomeric**  **band**  **(size)** |  |
| **1** | **4.0**  + 0.9 | **39.8**  + 3.7 | M | 2 | M | 3 | S |  |
| **2** | **3.9**  + 0.8 | **40.0**  + 3.6 | M | 1 | L | 2 | S |  |
| **3** | **3.8**  + 0.8 | **41.2**  + 3.5 | M | 1 | M | 3 | S |  |
| **4** | **3.6**  + 0.8 | **44.5**  + 4.0 | M | - | L | 1 | - |  |
| **5** | **3.5**  + 0.7 | **36.3**  + 3.8 | M | - | L | 2 | S |  |
| **6** | **3.4**  + 0.6 | **45.1**  + 3.4 | S | 1 | L | 2 | S |  |
| **7** | **3.1**  + 0.8 | **32.4**  +5.2 | L | - | L | 2 | M | 5S + 45S rDNA site in the  distal region of the short arm |
| **8** | **3.0**  + 0.8 | **40.2**  +5.6 | S | 1 | L | 1 | L | 5S + 45S rDNA site in the  distal region of the long arm |
| **9** | **2.6**  + 0.5 | **37.6**  + 3.7 | L | - | M | 1 | S | 5S rDNA site in the  distal region of the long arm |
| **10** | **2.4**  + 0.5 | **44.9**  + 3.9 | M | - | L | 1 | M |  |
| **11** | **2.4**  + 0.4 | **39.7**  + 4.8 | S | - | L | 1 | L |  |
| **12** | **2.1**  + 0.4 | **39.9**  + 4.1 | S | - | L | 1 | S |  |
| **13** | **2.0**  + 0.4 | **45.3**  + 4.7 | M | - | M | - | L |  |

“centromeric index” – the ratio of the length of the short arm of the chromosome to that of the total chromosome; “s.d.” – standart deviation; “S” – small; “M” – middle; “L” – large.
